# Effects of metaverse-based career mentoring for nursing students: a mixed methods study

**DOI:** 10.1186/s12912-023-01323-8

**Published:** 2023-05-15

**Authors:** Yujeong Kim, Mi Young Kim

**Affiliations:** 1 College of Nursing, Research Institute of Nursing Science, Kyungpook National University, Daegu, Republic of Korea; 2 College of Nursing, Hanyang University, Seoul, Republic of Korea

**Keywords:** Career Choice, Mentoring, Virtual reality, Self-efficacy, Nursing students

## Abstract

**Background:**

There is a lack of studies on metaverse-based career mentoring for college students in both quantitative and qualitative research. This study aimed to examine the effect of metaverse-based career mentoring among nursing students and explore the experiences of mentors and mentees.

**Methods:**

This study used a mixed methods design using both a survey for collecting quantitative data and focus group interviews for a qualitative one. A total of 8 mentors and 43 mentees participated in the metaverse-based career mentoring program. The program covered eight career fields and was delivered across eight sessions of 60 min each, over six days. Career decision-making self-efficacy among mentees and platform and program satisfaction were measured before and after the program. Afterwards, 7 mentors and 12 mentees participated in the focus group interviews to investigate their experience of participating in the metaverse-based career mentoring program. Quantitative data were analyzed using descriptive statistics, paired t-test, Wilcoxon signed-rank test, and Mann-Whitney U test. The qualitative data underwent thematic analysis.

**Results:**

After the metaverse-based career mentoring program, mentees’ career decision-making self-efficacy increased significantly compared to the baseline level. From the mentor–mentee focus group interviews, three key themes were derived: (i) communicating frankly and openly, (ii) being satisfied with realistic communication and program functions, and (iii) expecting an even more optimized program.

**Conclusions:**

A metaverse-based career mentoring program for nursing students can have a positive effect on their career decision-making self-efficacy. In addition, in terms of education, it is helpful as a non-face-to-face medium and feeling a sense of reality, so it is expected that it will be beneficial in education by applying various contents in the future.

**Supplementary Information:**

The online version contains supplementary material available at 10.1186/s12912-023-01323-8.

## Background

With the demand for nurses increasing due to recent changes in the healthcare environment, such as the outbreak of a novel infectious disease, a growing number of chronic diseases, the growing share of the older population, and advances in medical technology, the career path of nursing graduates is becoming increasingly diversified [1].

Meanwhile, due to the COVID-19 pandemic, a considerable proportion of theoretical lectures and clinical training at healthcare facilities have been switched to non-face-to-face settings, with more departments placing restrictions on nursing students’ clinical training. Students no longer have sufficient opportunities to explore career paths by exchanging information about career and employment in meetings with peers or alumni [2]. This lack of information, necessary for career exploration, makes it more difficult for nursing students to make career decisions [3].

Career exploration refers to exploration regarding the external environment based on an understanding of internal factors, such as personality, aptitude, interest, and value for the purpose of career development, including career decision and preparation, employment, and post-employment adjustment [4]. Insufficient career exploration has been found to impair career decision-making and cause stress among nursing students [5]. This highlights the importance of providing active interventions for nursing students’ career exploration activities. As we have seen, prior research [6] has shown that career mentoring is helpful as a career exploration activity. However, in crises such as the COVID-19 pandemic, face-to-face mentoring in various fields is difficult due to time and physical limitations [2]. Therefore, it is necessary to make a methodological attempt to make career mentoring in multiple domains without being affected by the environment or situation.

In a previous study, career mentoring was found to be effective in nursing students’ career exploration activities [6]. Through career mentoring, an experienced mentor shares knowledge and experience with a mentee, providing them with professional guidance and emotional support [7], thus enhancing the mentee’s career decision-making self-efficacy, that is, beliefs and confidence that one can successfully perform career-related tasks [8]. Career self-efficacy was found to have a positive effect on students’ career path exploration, goal setting, and career-related decision-making [6]. Consequently, great importance is attached to career mentoring as a strategy to enhance nursing students’ career decision-making self-efficacy.

During the current prolonged COVID-19 pandemic, where we have seen a paradigm shift of education from traditional classroom learning to online learning, the need to change the modality of career mentoring to increase students’ activities has also been raised [9]. In particular, the metaverse is increasingly attracting attention for overcoming the shortcomings of existing videoconferencing tools and implementing smooth interactions with less spatiotemporal constraints [10]. Non-face-to-face methods using existing videoconferencing tools, such as Zoom, have weak interaction and no spatial mobility, making it difficult to create a sense of spatial presence among participants [11]. In contrast, the metaverse is gaining attention as an alternative capable of overcoming the limitations of existing career education resources, owing to its advantages of enhancing interactivity and immersion while maintaining anonymity during counseling; it provides a learning environment that facilitates immersive interactivity using avatars [10].

Previous studies have applied metaverse in materials science education as a problem-based learning medium [12], university online classes [13], and high school career education programs [9]. “Metabus-based” was mainly used as a game or entertainment function or as a one-off event. There are a few cases in which metaverse has been used for small-group counseling, such as career mentoring. There is still a lack of studies on metaverse-based career mentoring for college students in both quantitative and qualitative research. It would be appropriate to conduct mixed research that includes both quantitative and qualitative research methods to derive advantages and disadvantages that are difficult to find in quantitative analysis, including interviews for qualitative research.

Against this background, this study aimed to examine the effect of metaverse-based career mentoring among nursing students. To this end, two objectives were set:

1) Using quantitative research methods through questionnaires, mentees’ self-efficacy in career decisions and the satisfaction of mentors and mentees after metaverse-based career mentoring are confirmed.

2) A qualitative exploration of mentors and mentees’ experiences with metaverse-based career mentoring using focus group interviews.

## Methods

### Design

This study used a mixed methods design using both a survey for collecting quantitative data and focus group interviews for a qualitative one. This study was conducted in a convergent parallel manner by collecting quantitative and qualitative data, analyzing the results separately, and deriving an overall interpretation during the discussion process [14].

### Study population

Participants consisted of mentors and nursing student mentees. Mentors were licensed nurses who had at least one year of working experience in various fields of nursing and were capable of providing career counseling to nursing students in their respective fields. The sampling strategy was convenience sampling. They were recruited through personal recommendations. Mentees were undergraduate nursing students aged 18 years and over, who were recruited through social network service announcement in two nursing schools. The sample size was calculated using the G Power 3.1.9.4 to determine the minimum sample size for performing paired t-test with the following parameters: a two-sided significance level (α) of 0.05, a statistical power (1-β) of 0.80, and a median effect size of 0.5. The minimum sample size was 34 participants. Those who understood the purpose of the study explained in the recruitment announcement and agreed to voluntarily participate were enrolled. An experience of at least six months in using Zoom was a prerequisite for both mentors and mentees. Eight mentors and 43 mentees were finally selected to participate in the metaverse-based career mentoring program developed in this study. The pre- and post-intervention questionnaire surveys were administered to all participants at the baseline and after the program. In the qualitative study, data collection continued until data satiation was reached and no new information emerged from the interviews. As a result, 19 participants (one mentor group of seven mentors and two mentee groups of six mentees each) participated in the focus group interviews.

### Measures

#### Quantitative research

As the general characteristics of mentors and mentees, gender, age, school year (mentees) or career (mentors), nursing major satisfaction, videoconferencing fatigue, virtual reality (VR) content experience, and metaverse experience were examined. The mentees’ career decision-making efficacy was measured before and after the career mentoring. To compare platform satisfaction between Zoom and the metaverse, participants’ satisfaction with both platforms was assessed before and after conducting the metaverse-based career mentoring program, respectively. In addition, the satisfaction with career mentoring was assessed in both mentors and mentees after the program administration. The quantitative data were collected from February 16 to 26, 2022.

##### Career decision-making self-efficacy

Career decision-making self-efficacy among mentees was assessed before and after the administration of career mentoring and the scores were compared. It was measured with the Career Decision-making Self-Efficacy Scale Short Form (CDSES-SF) developed by Betz and Voyten [15]. The CDSES-SF consists of 25 items that cover five domains: self-appraisal, occupational information, goal setting, planning, and problem-solving. Each item is rated on a five-point Likert scale (1 = strongly disagree, 5 = strongly agree). A higher total score indicates a higher level of career decision-making self-efficacy. Cronbach’s α of the tool was 0.93 at the time of development and 0.95 in this study.

##### Platform satisfaction

For a quantitative assessment of platform satisfaction, participants’ satisfaction with Zoom and the metaverse were measured before and after conducting the metaverse-based career mentoring program in both mentors and mentees. For platform satisfaction, immersion, social presence, sense of being together, interactivity, fatigue, emotional expression, and overall platform satisfaction were measured with reference to previous studies [9, 16–19].

Immersion was assessed by measuring the scope of influence of the levels of immersivity, virtual reality, and interactivity with avatars (including motion and voice interactions) on immersion. The immersion scale consists of six items, with each item rated on a 10-point scale (1 = very dissatisfied, 10 = very satisfied), where a higher total score indicates a higher immersion in the program. The reliability of the scale (Cronbach’s α) in this study was 0.90.

Social presence was measured using the 10-item social presence scale developed by Weidlich and Bastiaens [16]. Examples of items are “This program feels as if we communicate face-to-face” and “In this program, I feel as if all the group members are real persons.” Each item is rated on a seven-point Likert scale (1 = strongly disagree, 7 = strongly agree), where a higher total score indicates a higher level of social presence. Cronbach’s α was 0.90 at the time of development and 0.95 in this study.

Sense of being together refers to the sense of being together with other learners in the learning space. In this study, it was measured with the instrument developed by Kim et al. [17]. Each of the six items is rated on a seven-point Likert scale (1 = strongly disagree, 7 = strongly agree), where a higher total score indicates a higher level of the sense of being together with other learners in the same space. Cronbach’s α was 0.72 at the time of development and 0.83 in this study.

Interactivity was measured with a tool developed by Oh [18] and modified and supplemented by Park and Kang [19]. It consists of six items, and each item is rated on a seven-point Likert scale (1 = strongly disagree, 7 = strongly agree), where a higher total score indicates a higher level of perception of interactivity on the platform. Cronbach’s α was 0.89 in the study by Park and Kang [19] and 0.94 in this study.

Fatigue was measured with a one-item scale for overall fatigue from platform use. The item is rated on a five-point Likert scale (1 = very low, 5 = very high). A higher score indicates a higher level of fatigue from platform use.

Emotional expressivity was measured with a one-item scale that measures the level of expressing one’s own opinion or feeling in the platform concerned. It is rated on a five-point Likert scale (1 = hesitant, 5 = not hesitant at all). A higher score indicates a higher level of freedom in expressing feelings on the platform.

Overall platform satisfaction was measured with a tool developed by Lim et al. [9]. It consists of five items: novelty, realism, ease of use, stability, and usability. Each item is rated on a five-point Likert scale (1 = strongly disagree, 5 = strongly agree). A higher score indicates a higher level of platform satisfaction. Cronbach’s α was 0.81 in this study.

##### Satisfaction with metaverse-based career mentoring program

Satisfaction with the metaverse-based career mentoring program was assessed through separate surveys with mentors and mentees. For program satisfaction, counseling satisfaction, utility, ease of use, and intention to use were measured with reference to previous studies [20–25].

Counseling satisfaction was measured using the Client Satisfaction Questionnaire developed by Larsen et al. [20] and modified and supplemented by Kim [21]. It includes client and counselor sections, consisting of eight items each. Each item is rated on a seven-point Likert scale (1 = strongly disagree, 7 = strongly agree), where a higher total score indicates a higher level of counseling satisfaction. Cronbach’s α was 0.96 for the clients in Kim’s [21] study, and 0.92 for the clients and 0.98 for counselors in this study.

Utility was measured using the tool developed by Qiao and Han [22] for virtual reality contents, and modified and supplemented by Oh [23] for metaverse contents. It consists of three items, and each item is rated on a five-point Likert scale (1 = strongly disagree, 5 = strongly agree). A higher total score indicates a higher perception of utility of the metaverse program. Cronbach’s α of the tool was 0.91 in Oh’s [23] study and 0.82 in this study.

Ease of use was measured with the tool developed by Qiao and Han [22] for virtual reality contents, and modified and supplemented by Oh [23] for metaverse contents. It consists of three items, and each item is rated on a five-point Likert scale (1 = strongly disagree, 5 = strongly agree). A higher total score indicates a higher perception of ease of use of the metaverse program. Cronbach’s α of the tool was 0.95 in Oh’s [23] study and 0.66 in this study.

Intention to use refers to the willingness to use the metaverse program in the future. It was measured using the tools developed by Venkatesh and Davis [24] and Choi et al. [25], and modified and supplemented by Oh [23] for the metaverse. It consists of three items, and each item is rated on a five-point Likert scale (1 = strongly disagree, 5 = strongly agree). A higher total score indicates a higher perception of ease of use of the metaverse program. Cronbach’s α of the tool was 0.90 in Oh’s [23] study and 0.95 in this study.

#### Qualitative study

After completing the career mentoring program, focus group interviews were conducted only with those who completed the survey and wanted to participate in the interviews. One session of 60 to 70 min was held for each of the three focus groups (one mentor group of seven mentors and two mentee groups of six mentees each). Data were collected from February 21 to 26, 2022. In order to draw out free and diverse opinions from the interviewees, the researcher experienced in focus group interviews conducted the sessions in a comfortable atmosphere in a metaverse setting.

Focus group interviews were conducted using a semi-structured questionnaire. The key question was “How did mentors and mentees experience metaverse-based career mentoring for nursing students?” More specifically, this issue was addressed by the following detailed questions: “Tell me about your experience with metaverse-based career mentoring,” “What feelings, emotions, and thoughts did you have during metaverse-based career mentoring?,” “What did you like about your experience with metaverse-based career mentoring?,” “What are the merits and demerits of metaverse-based career mentoring compared to other face-to-face or non-face-to-face mentoring styles?,” and “What should be improved in metaverse-based career mentoring?” During the interview process, interviewees were encouraged to freely talk about any additional questions they thought were necessary or anything else they wanted. Upon completion of the interviews, any doubts about the content were clarified via phone.

### Procedure

#### Development of metaverse-based career mentoring program for nursing students

This study was conducted according to the Analysis-Design-Development-Implementation-Evaluation (ADDIE) model [26] to develop a metaverse-based career mentoring program for nursing students.

First, in the analysis phase, the career-related mentoring needs of nursing students were analyzed. To this end, a survey was conducted from January 24 to 28, 2022, with undergraduates enrolled in a nursing college. A total of 46 students participated in the preliminary needs survey, and the nursing field most desired to receive career mentoring was overseas nurses (30.5%), followed by pharmaceutical company researchers (28.3%), clinical nurses (23.9%), and nursing-related government officials (17.3%). In addition, previous studies regarding career mentoring programs for nursing students [6] were reviewed with a focus on the program structure and operation method.

Second, in the design and development phases, the career fields for applying the mentoring program were selected by reflecting the students’ needs, and the program was structured to have eight sessions on six days. The duration of each session was set to 60 min to give sufficient time for the mentor’s explanation of the relevant career field and answers to mentees’ questions. The program contents are outlined in Table 1.

**Table 1:** Metaverse-based Career Mentoring Program (*N* = 46)

Category	Activities	Time (min)	Contents	Methods	Materials
Before program	Orientation	30	□ Introduction for study □ Written consent	Explanation	Zoom
	Pre-test	15	□ General characteristics □ Platform (Zoom) satisfaction □ Career decision-making self-efficacy	Survey	Online
Career mentoring program	1 Ward nurse	60	□ Introduction related to career □ Main business □ Career-related merits/demerits □ Career-related preparations □ Questions and answers	Group mentoring	Metaverse
	2 Special unit nurse	60			
	3 Overseas nurse	60			
	4 Corporate health manager	60			
	5 School nurse	60			
	6 National/public corporation	60			
	7 Pharmaceutical company researcher	60			
	8 Nursing government official	60			
After program	Post-test	15	□ Platform (Metaverse) satisfaction □ Satisfaction with metaverse-based career mentoring □ Career decision-making self-efficacy	Survey	Online
	Orientation	30	□ Introduction for interview	Explanation	Metaverse
	Focus group interview	60 –70	□ Core questions & Detailed questions	Interview	

Prior to the program implementation, a videoconferencing orientation session was given to each group (one mentor and two mentee groups) to explain the purpose and procedure of this study. Only after receiving online consent from each of those who wished to voluntarily participate, a preliminary 15-minute questionnaire survey was conducted.

Mentors discussed the date and time among the six days when they were available, which was then reflected in the program planning; they were given a 15-minute preparation time for explaining the major tasks, strengths and weaknesses, and career-related preparations in their respective career fields.

Mentees were allowed to check the career mentoring program schedule and write down the first and second preferences for the subgroup they wanted to participate in. Based on the application details of the mentees, the maximum number of mentees per group was set at eight. Before applying the career mentoring program in this study, a pilot test was performed on three junior and senior students of a nursing school. The metaverse platform used in this study was the Metaforest developed by YATAV Inc., which was tested preliminarily by using it in advance. As a result, problems that may occur in the mediation process, such as avatar operation and conversation participation, could be identified, and a detailed manual was prepared and supplemented.

Third, in the implementation phase, the actual metaverse-based career mentoring program was performed from February 21 to 26, 2022. A week before participating in the program, the research assistant sent the mentors and mentees their respective metaverse program installation files and manuals to be used in this study, by e-mail with the instruction to install them in advance and try them out. The researcher explained the simple operation method to the mentors and mentees connected to the metaverse for about 20 min before the start of each mentoring program session, and instructed them to check for any problem in operation. The subgroup career mentoring was conducted over six days, and an online post-survey was conducted on completion of the program. At the beginning of the program, participants were recruited among those who wanted to be voluntarily interviewed about their participation experience after the program’s end, and interview dates were set for each group of mentors and mentees.

Lastly, in the evaluation phase, pre- and post-intervention online surveys were conducted, using a self-report questionnaire, at the baseline and immediately after the program, respectively, in order to evaluate the program efficacy. The focus group interviews regarding experiences of participating in the program were directly conducted by the researcher, who was proficient in conducting qualitative research.

### Data analysis

Quantitative data were analyzed using SPSS 23.0 program. The participants’ general characteristics were analyzed using descriptive statistics. Testing for normality was performed to identify the pre-post differences in variables before and after the metaverse-based career mentoring. A paired t-test was applied to analyze mentees’ social presence, sense of being together, and overall platform satisfaction, which met normal distribution, and Wilcoxon Signed-Rank test and Mann-Whitney’s U test were applied to analyze the remaining variables, which failed to meet normal distribution.

For qualitative data, a thematic analysis was performed, in which main themes were extracted by identifying the relationships between key concepts from the transcripts of each focus group interview, to explore the experiences of participants in the metaverse-based career mentoring program. The researcher listened to each transcript repeatedly, extracted and coded meaningful words, phrases, and sentences while carefully reading the statements made by the interviewee, grouped them into similar ones, and finally extracted the main theme.

### Ensuring rigor in qualitative research and researchers’ preparations

In order to ensure the rigor in qualitative research, credibility, fittingness, auditability, and confirmability were checked, as suggested by Guba and Lincoln [27]. First, credibility was ensured by recording the interview content and literally transcribing it and extracting concepts by faithfully describing the terms used by the interviewee. Furthermore, to obtain results free from the researcher’s prejudices or biases, neutrality was maintained throughout the interview and result analysis process. Second, to ensure fittingness, three participants were requested to check whether the contents described and analyzed by the researcher were consistent with their experiences. Additionally, feedback regarding the appropriateness of the obtained results was received from a nursing professor with extensive experience in conducting qualitative research. Third, to ensure auditability, the analysis procedure was explained in detail in the methods section of this study, and participants’ statements were directly quoted in the results for the reader to check the researcher’s interpretation or analysis. Fourth, confirmability was ensured by the strict observance of reliability, suitability, and auditability.

To ensure reliability of researchers who analyze qualitative data and train them to reflect on the results, all researchers have taken lectures on qualitative research at graduate courses and special lectures in related conferences. The researchers have also conducted several qualitative researches. In addition, since the researchers worked as professors in nursing colleges for more than 8 years and continuously conducted career counseling for nursing students, we were able to have sensitivity in understanding the career mentoring experience of nursing students. The researchers were excluded as much as possible so that the experiences and opinions of the participants could be fully reflected and the researcher’s opinions or prejudices could not affect the analysis of the results and maintain a neutral role.

### Ethical considerations

The study was approved by Kyungpook National University’s Institutional Review Board (no. KNU-2022-0020). To ensure participants’ anonymity, data points collected by the researcher were randomly assigned with numbers to minimize the risk of exposure. The informed consent was obtained from all participants for study participation. The study was conducted following the Declaration of Helsinki.

Prior oral consent was obtained online from each participant in the qualitative study, and an online written consent form was received on the interview day after explaining the purpose of the study and interview procedure again. All interviews were recorded, and the interviewees were informed that their privacy would be protected, and all data would be processed as anonymized or coded data to prevent identity exposure. The participants were told that they could withdraw from the study at any time if they no longer wished to participate, without receiving any benefits from participation or disadvantages from nonparticipation. Ethical issues regarding plagiarism, informed consent, misconduct, data fabrication and/or falsification, double publication and/or submission, and redundancy were observed by the author.

## Results

### Effects of applying metaverse-based career mentoring program

#### Participants’ general characteristics

Of the 46 participants, 38 were mentees with the following general characteristics: 92.1% were female students; their mean age was 20.79 years; sophomores had the highest percentage (42.1%); 50% had high nursing major satisfaction; 71.1% had virtual reality experience; and 52.6% had metaverse experience. Mentors (n = 8) were all women; their mean age was 31.38 years and mean career length was 4.69 years; 87.5% had high major satisfaction; 62.5% had no virtual reality experience, and 50.0% had metaverse experience (Table 2).

**Table 2:** General Characteristics of Mentees and Mentors (*N* = 46)

Variables	Categories	Mentee (n = 38)	Mentor (n = 8)
		n (%), M ± SD	
Gender	Male	3 (7.9)	-
	Female	35 (92.1)	8 (100%)
Age (years)		20.79 ± 1.40	31.38 ± 4.69
Grade	2	16 (42.1)	-
	3	8 (21.1)	
	4	14 (36.8)	
Total career (years)		-	4.69 ± 3.67
Major satisfaction	≤Moderate	11 (28.9)	1 (12.5)
	High	19 (50.0)	7 (87.5)
	Very high	8 (21.1)	-
Virtual reality experience	Yes	27 (71.1)	3 (37.5)
	No	11 (28.9)	5 (62.5)
Metaverse experience	Yes	20 (52.6)	4 (50.0)
	No	18 (47.4)	4 (50.0)

#### Difference in career decision-making self-efficacy before and after the metaverse-based career mentoring program

A comparison of career decision-making self-efficacy in mentees before and after the metaverse-based career mentoring program showed that career decision-making self-efficacy significantly increased after participation in the program compared to the baseline level (Table 3).

**Table 3:** Comparison of Mentees’ Career Decision-making Self Efficacy between Pre-test and Post-test (*N* = 38)

Variables	Pre-test	Post-test	t (p)
	M ± SD		
Career decision-making self-efficacy	3.57 ± 0.55	3.96 ± 0.58	5.89 (< 0.001)

#### Satisfaction of mentees and mentors

Table 4 outlines the results of comparison between Zoom satisfaction from prior experience and metaverse satisfaction after using the proposed program in mentees and mentors. Mentees obtained significantly higher scores for the metaverse platform in immersion, sense of being together, interactivity, and emotional expressivity. Fatigue was significantly higher for the Zoom program compared to the metaverse platform. No significant differences were observed in the remaining items. Mentors scored significantly higher for the metaverse platform versus the Zoom program in emotional expressivity, and showed no significant differences in the other items.

The satisfaction of mentees and mentors with the metaverse-based career mentoring program showed that the level of counseling satisfaction was 8.66 ± 1.46 for mentees and 6.90 ± 1.92 for mentors. In the mentees, utility was 3.65 ± 0.79, ease of use was 3.57 ± 0.75, and intention to use was 3.64 ± 1.01. In mentors, utility was 3.29 ± 0.98, ease of use was 3.88 ± 0.73, and intention to use was 3.33 ± 1.17 (Table 4).

**Table 4:** Satisfaction of Mentees and Mentors (*N* = 46)

Variables		Mentee (n = 38)			Mentor (n = 8)		
		Pre-test (Zoom)	Post-test (Metaverse)	t, Z (p)	Pre-test (Zoom)	Post-test (Metaverse)	Z (p)
		M ± SD			M ± SD		
Platform	Immersion	6.53 ± 1.84	7.74 ± 1.91	2.59 (0.008)	6.63 ± 1.30	6.85 ± 2.17	0.85 (0.398)
	Social presence	4.47 ± 1.20	4.87 ± 1.41	1.22 (0.232)	4.30 ± 1.20	4.51 ± 1.61	0.68 (0.499)
	Social space	3.51 ± 0.91	4.36 ± 1.10	3.61 (0.001)	3.19 ± 0.49	4.06 ± 1.39	1.90 (0.058)
	Interactivity	4.64 ± 1.16	5.39 ± 1.47	2.47 (0.012)	4.67 ± 0.78	4.88 ± 1.40	0.42 (0.671)
	Fatigue	3.53 ± 0.92	2.55 ± 1.03	3.93 (< 0.001)	3.00 ± 0.76	3.00 ± 0.75	0.00 (1.00)
	Emotional expressivity	2.71 ± 1.11	3.66 ± 1.07	3.04 (0.002)	2.87 ± 0.64	4.13 ± 0.64	2.46 (0.014)
	Platform satisfaction	3.35 ± 0.56	3.62 ± 0.73	1.80 (0.081)	3.43 ± 0.45	3.50 ± 0.75	0.43 (0.671)
Program	Counseling satisfaction	-	8.66 ± 1.46	-	-	6.90 ± 1.92	-
	Utility	-	3.65 ± 0.79	-	-	3.29 ± 0.98	-
	Ease of use	-	3.57 ± 0.75	-	-	3.88 ± 0.73	-
	Intention to use	-	3.64 ± 1.01	-	-	3.33 ± 1.17	-

#### Experience of the metaverse-based career mentoring program

A total of 46 codes were drawn by classifying significant phrases and sentences stated by each of the mentors and mentees about their experience with the metaverse-based career mentoring program. The codes with similar contents were grouped together and classified into 28 items of more general forms, which were then subjected to further combination and separation. As a result, three main themes and five categories were drawn. The main themes thus drawn were “candid interviews without constraints,” “satisfaction with realistic talks and program functions,” and “expectation of an even more optimized program.” The qualitative content analysis results are summarized in Table 5.

**Table 5:** Qualitative Content Analysis Results

Categories	Sub-categories	Subjects
Candid interviews without constraints	• Avatar-mediated anonymity without the burden of identity disclosure	Mentees & Mentors
Satisfaction with realistic talks and program functions	• Gratification to have new and accurate information about career path • Boosted concentration owning to a realistic program • Satisfaction with application of the metaverse to the mentoring platform	Mentees Mentees & Mentors Mentees & Mentors
Expectation of an even more optimized program	• Insufficiencies of some program functions • Demand for affinity-enhancing functions	Mentees & Mentors Mentees & Mentors

#### Theme 1: candid interviews without constraints

##### Avatar-mediated anonymity without the burden of identity disclosure

Mentees noted that by anonymously participating in the career mentoring program via an avatar they selected, their face was not exposed to others, so they did not feel burdened while participating. Mentors also mentioned that participation without exposing their faces made them answer the mentees’ questions more candidly and comfortably; they could play their mentor role more honestly because mentees asked anything they wanted to know without any constraints. Since the existing online programs expose at least one’s face or some identifiable cues, timid participants face as much psychological pressure as in any offline program. However, all participants felt free to ask any questions in the metaverse-based career mentoring program, including sensitive ones, which made them participate in the program more actively.

#### Theme 2: satisfaction with realistic talks and program functions

##### Gratification to have new and accurate information about career path

Mentees stated that listening to various onsite experiences and stories from mentors helped them in their career exploration. They obtained a wide variety of information different from what they had come across through career lectures or Internet queries, and were satisfied with their participation in the program because they could obtain candid and accurate information about the matters they had been curious to know; these included preparations necessary for a given field, major tasks after employment, welfare matters, and promotion. They also mentioned that it was an opportunity to broaden their perspective of nursing-career paths.

##### Boosted concentration owning to a realistic program

Participants admitted that the avatars felt so realistic and lively that they even thought they were not virtual characters. They also found that although the metaverse was a virtual space, they could concentrate better through it than through video call or videoconferencing, by talking in a space created like a real-world conference room; it gave them a sense of realism.

##### Satisfaction with application of the metaverse to the mentoring platform

Some participants encountered the metaverse for the first time, and although it took some time to adapt, they said that the platform as a mentoring program was fresh compared to the existing offline and online programs. They were satisfied with the metaverse program because it allowed people to talk to each other via avatars in a virtual space, react to others by making facial expressions or movements, and chat; it was free from spatial and temporal restraints and could be applied to various spatial settings, in addition to lecture rooms. Both mentors and mentees said that they could participate with interest.

#### Theme 3: expectation of an even more optimized program

##### Insufficiencies of some program functions

Some participants found it regretful not being able to decorate their own avatars as much as they wanted, and some functions such as motions and facial expressions lacked diversity. In addition, it took some time to install the program or get familiarized with basic functions, which made the process of prior preparations somewhat cumbersome.

##### Expecting for affinity-enhancing functions

Participants regretted that they could not form a close affinity during the career mentoring program due to lacking information on the mentors and other mentees. Moreover, it was difficult for a mentor to check whether the mentees were listening to their words, including what emotions or responses they were displaying through the motions or facial expressions shown by their avatars. In the process of mentees writing questions in the chat box to avoid overlapping voices and the mentor answering them verbally, the mentor felt like performing a solo show. She expressed her wish that the affinity among participants could be enhanced more easily through functions, such as boosting the interactions between a mentor and her mentees as well as among all participants, or checking others’ reactions.

## Discussion

This study aimed to provide an understanding of the effects of metaverse-based career mentoring for nursing students, including the related experiences of mentors and mentees, applying a mixed research method. In this study, career decision-making self-efficacy was significantly increased after the administration of career mentoring, which is consistent with the finding of a previous study [28] in which workers’ job mentoring was provided to female college students. A higher career decision-making self-efficacy is known to be associated with lower anxiety level and higher career decision-making ability through more active career exploration activities [29]. In 2019, the turnover rate of novice nurses in Korea was as high as 45.5% [30]. This highlights the importance of devising a personalized path and demonstrating professional abilities during the undergraduate years, through a systematic preparation process to adapt to the role of a nurse after graduation. Given that counseling programs for undergraduates, such as career mentoring aiming at boosting career decision-making self-efficacy, have an important impact on career choice [31], it is necessary to deliver various programs that instill self-initiative and confidence in the nursing students regarding their career path.

A comparison of the participants’ platform satisfaction between Zoom and the metaverse showed that the mentees perceived significantly higher levels of platform satisfaction with the metaverse than with Zoom, for the variables “immersion,” “interactivity,” “emotional expressivity,” and lower level of “fatigue.” This may be due to the fact that the mentees who participated in this study fall in the age range of Generation Z members, and are, therefore, familiar with the Internet and digital devices since their childhood, with their affinity for the metaverse presumed to be high [32]. In addition, the Korean Generation Z individuals reportedly use metaverse platforms, such as Roblox, more frequently than social media platforms [33]. In the metaverse, users are directly connected to a virtual world via their avatars without being interrupted from the real world, which ensures high immersion; they can also freely interact and communicate with one another without any spatiotemporal constraints, with their identity perfectly protected [32], which is presumably perceived as an advantage over Zoom. Metaverse has been reported to cause significantly less fatigue compared to motion-restricted webcam or face-to-face meetings [34]. The college being a space mostly frequented by young people in their twenties, such advantages of the metaverse platform will serve as the basis for expanding its application to education, in addition to virtual mentoring [35].

However, mentors showed a significantly higher level of platform satisfaction with metaverse than with Zoom only in emotional expressivity, and no significant differences with Zoom were observed in the other variables. Moreover, their counseling satisfaction was significantly lower than that of mentees after the administration of career mentoring. This may be ascribed to the difficulties in playing their role of attending to the atmosphere of mentoring and counseling due to constraints in interactions with the mentees; the use of avatar characters made it more difficult to understand the spontaneous responses of the mentees during the counseling process compared with face-to-face or videoconferencing interactions. This lack of advantages of the metaverse over Zoom in immersion or fatigue may have led to rather low counseling satisfaction among the mentors. Conversely, with anonymity guaranteed, the mentees could ask more sensitive questions and the reactions of other participants were not very important to them, which may explain the difference in platform or program satisfaction between mentors and mentees. Therefore, in order to achieve high-level communication and interaction activities, which involve catching the inter-participant reactions, it is necessary to develop various communication patterns of the avatar, including the eye direction, body posture, and facial expressions that recognize each other’s presence in avatar characters [32]. It is also necessary to develop a hybrid-type career mentoring program that combines the advantages of the metaverse and face-to-face meetings, comparing the effects.

The participants in this study perceived a sense of being together in the same counseling space, but felt that it was insufficient to form close affinity. This lack of affinity building was ascribed partly to limitations in the implementation of the metaverse program, and partly to the lack of mutual information due to anonymity. The persistence of the COVID-19 pandemic has accelerated the transition to a contact-free society, and the metaverse provides a novel social space to enable connection that allows people to meet and communicate among themselves [36]. In the field of education as well, some hold the view that metaverse-based education enables interactions corresponding to face-to-face education [37], but there is also the opinion that the social connection in the metaverse is weaker than that of the real world [36]. There is criticism that research on the current metaverse platforms is in its inception phase, and the defendants of metaverse emphasize only its novel experiential aspects, turning a blind eye on the deep mental exchanges between participants [38]. To overcome these limitations, a variety of learning activities should be designed in a way to induce efficient expansion of the educational space. To this end, university faculties must have the confidence and skills to use the metaverse and build media literacy competency. In addition, extensive studies should be conducted with the aim of demonstrating the effects of metaverse-mediated learning and counseling.

This study was significant in that it delivered metaverse-based career mentoring to enhance the career decision-making self-efficacy of nursing students. The mentees’ satisfaction with the metaverse platform was higher compared with existing videoconferencing platforms in immersion, sense of being together, interactivity, fatigue, and emotional expressivity. Regarding the limitations of this study, it may be pointed out that since career mentoring was administered only once to nursing students in two nursing schools of Korea, it is difficult to generalize the study results. Moreover, since only single-group testing was performed to analyze the effects of career mentoring, it is necessary to test the effectiveness of metaverse-based career mentoring more objectively through randomized controlled trials.

## Conclusion

In this study, metaverse-based career mentoring was found to be effective in enhancing nursing students’ career decision-making self-efficacy. Through the focus group interviews, it was found that participants experienced positive program functions, such as candid interviewing, facilitated concentration, and acquisition of new information on career path, also lacking certain functions, such as insufficiencies in building a close affinity among participants. In order to check whether career decision-making self-efficacy enhanced through the career mentoring program will be maintained, a longitudinal study needs to be conducted to determine individuals’ satisfaction with the career decision-making at the time of graduation. In this context, studies focusing on the development and interventional effects of a hybrid-type career mentoring program that combine the advantages of the metaverse and face-to-face counseling should be conducted.

## Electronic supplementary material

Below is the link to the electronic supplementary material.

Supplementary Material 1

## Data Availability

The data that support the findings of this study are available from the corresponding author upon reasonable request.
