# Supplementary material for: Effects of metaverse-based career mentoring for nursing students: a mixed methods study
Source: BMC Nurs. 2023 May 15;22:160. doi: 10.1186/s12912-023-01323-8 (PMC10183309; doi:10.1186/s12912-023-01323-8)
Supplement: Supplementary file 1 — Supplementary Material 1 [file 12912_2023_1323_MOESM1_ESM.docx]

Supplementary material

RESULTS

Theme 1: Candid interviews without constraints

Avatar-mediated anonymity without the burden of identity disclosure

*To start off, I think the best thing was anonymity, which helped overcome restraints in counseling, where one’s own weaknesses should be revealed and sensitive questions need to be addressed, especially by a timid person. I enjoyed it most that I felt no hesitation or burden. Especially when a lot of people gathered, I would have been rather reserved, thinking whether I was asking in the right way or I could ask a certain question, but I did not feel such burden.* (Mentee 7)

*I seem to enjoy asking questions. There are questions difficult to ask face-to-face, and there are also questions that require some caution. Then I ask myself — Can I ask this question? What would she think of me? Some matters like annual earnings are somewhat difficult to address in person. So, I think it was an advantage to be able to ask a little more frankly about something like that.* (Mentee 11)

*Anonymity is definitely an advantage. It was great to be able to address things I was curious to know about, especially something sensitive to talk about in gatherings, such as junior nurse bullying, promotion, annual earnings, and turnover, and other petty topics without any reservations.* (Mentor 1)

Theme 2: Satisfaction with realistic talks and program functions

Gratification to have new and accurate information about career paths

*When I was attending lectures on the Introduction to Nursing or similar classes, I was aware that there are various career paths for nurses. In fact, however, I never asked what career paths there were other than clinical nursing. It was somewhat difficult for me to make up my mind to attempt a specific path since nursing paths are diversified. It was very nice to meet nurses working on-site and ask them questions and listen to their answers, which is a rare opportunity for a student.* (Mentee 9)

*I think we need a lot more mentoring like this. I can follow the path of a nurse, but I want to know a lot about other nursing fields. In this regard, I think mentoring was of great help to me. Since my vison was broadened, I hope that other mentoring can also be provided frequently through metaverse.* (Mentee 11)

*I am glad I could tell the truth about low salaries in public corporations. In fact, it is the kind of information that cannot be gained through official channels, and I think it is something that we can find from what is circulating on the Internet, but nothing more detailed. I thought I could bring up more such covert and candid stories.* (Mentor 2)

Boosted concentration owing to a realistic program

*Although the face was not shown directly, the shape of the mouth moving or body in motion while speaking made me forget that it was a character. It was fun to see the characters showing their motions and expressions, and it helped me focus a little bit better.* (Mentee 4)

*In a Zoom classroom, I do not feel like I am attending the class. It remains unnoticed when I do something else, and even when my face is clearly visible, I am looking down and doing something else. In this program, however, I watch myself entering the lecture room, and feel as if I am entering a real lecture room. Sitting in my chair to follow the lecture gives me a feeling that I am really attending the class.* (Mentee 12)

*I often had the feeling that we were together. I must have focused on such visual aspects. As I continued to watch the screen, I got the feeling that we were really together. I think that was also due to the fact that the mentees of my group enthusiastically asked so many questions.* (Mentor 4)

Satisfaction with application of the metaverse to the mentoring platform

*I felt much less fatigue compared to a Zoom session. The screen showing me sitting in a chair made me feel like being mentored in a real gathering. When operating a program with a limited number of attendees, the metaverse is advantageous because time and space constraints are reduced. I think the biggest advantage of the metaverse is anonymity, and I believe it would be great when delivering psychological counseling at hospital or in school.* (Mentee 11)

*There were times when I wore a mask because I did not want to reveal my face in a Zoom session. In this program, however, with my avatar conveying my expressions on my behalf, it felt like I was participating in group counseling.* (Mentee 9)

*It struck me as new to be able to share the feeling of being together in a forest or other screens in the metaverse, in environments that cannot be realized in a classroom. If there was an opportunity, I thought it would be nice and refreshing to have group counseling in a metaverse environment with a bonfire.* (Mentor 5)

Theme 3: Expectation of an even more optimized program

Insufficiencies of some program functions

*In the metaverse, avatars have only limited expressions. One student keeps nodding, but it is impossible to see varied forms of each expression, and it makes you wonder if the student is listening to the conversation well. Also, I wanted to get the feeling that we were interacting with each other by hearing reactive tokens, so I asked the students to turn on the microphone and mention any reactive tokens. Unfortunately, it did not work because the sounds were entangled, and they had to go mute again. Also, in the metaverse setting, there was a forest behind the door, and I wanted to wrap up the session by lighting a bonfire. As we were set to go outside around the end of talks, there was a pell-mell of individuals running around. I thought it would take 10 minutes to move once, and there was again disorderly sitting and standing. If all participants familiarize themselves with the operation mechanism, it would improve bit by bit. (*Mentor 5)

*I thought that it would be easier to immerse oneself if avatar characters were more natural. Not only facial expressions, but also upper body motions can be recognized; the computer will naturally catch nodding of the head, which will improve immersion. And I wished that the audio would not be interrupted.* (Mentor 7)

Expecting for affinity-enhancing functions

*It was a bit difficult to tell if the person on the other end was listening to me while I was speaking. It would be nice to have something like an interaction button to ensure that others are listening when I am talking. It seemed that there was a face recognition function that allowed expression changes, but I thought that it would be better for immersion to have a configuration that makes the expressions more clearly visible. (Mentee 5)*

*I also felt like being alone. During a session, I would wonder whether it was a live broadcast on Instagram. It was difficult to hear when the voices got entangled, so students left questions in the chat box and I answered them. That may be the reason why I felt that way a lot. Hmm... The basics of a meeting or consultation are mostly conveyed through words, and non-verbal parts add aspects such as emotions. I think a faithful compliance with the basics to make the voice transmitted clearly like a landline call or Zoom would definitely increase the usability.* (Mentor 3)
